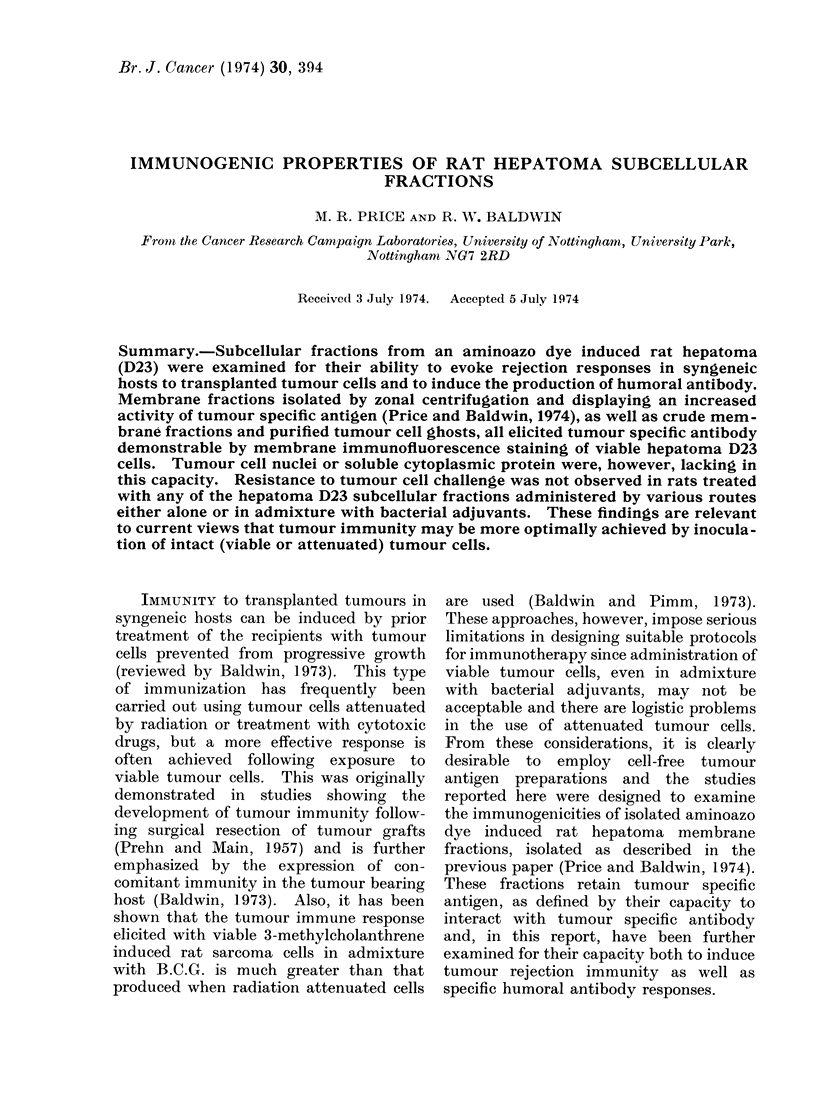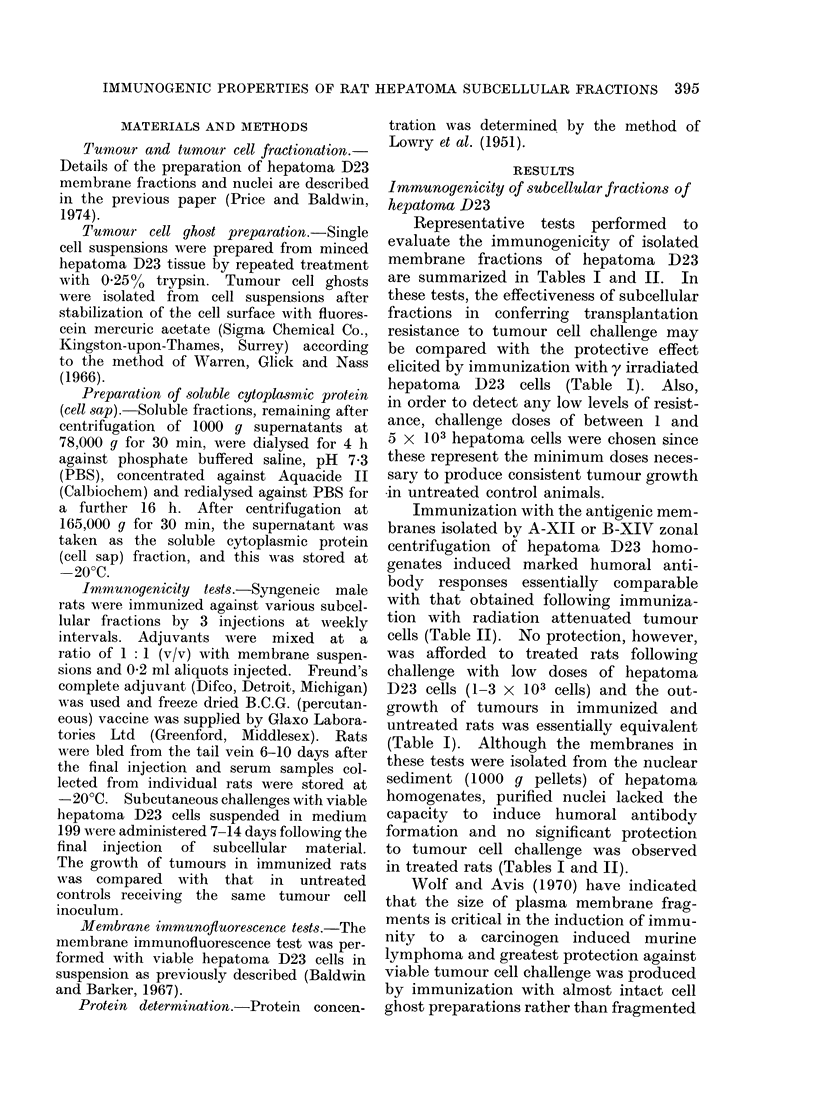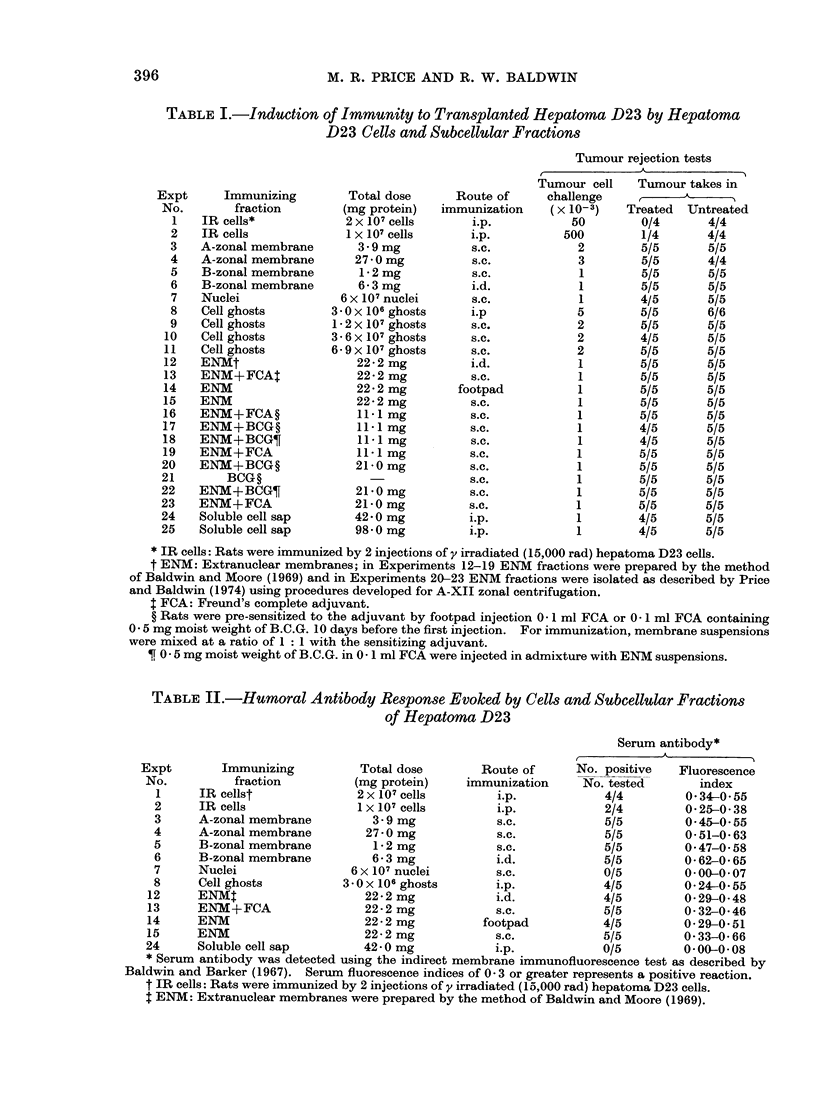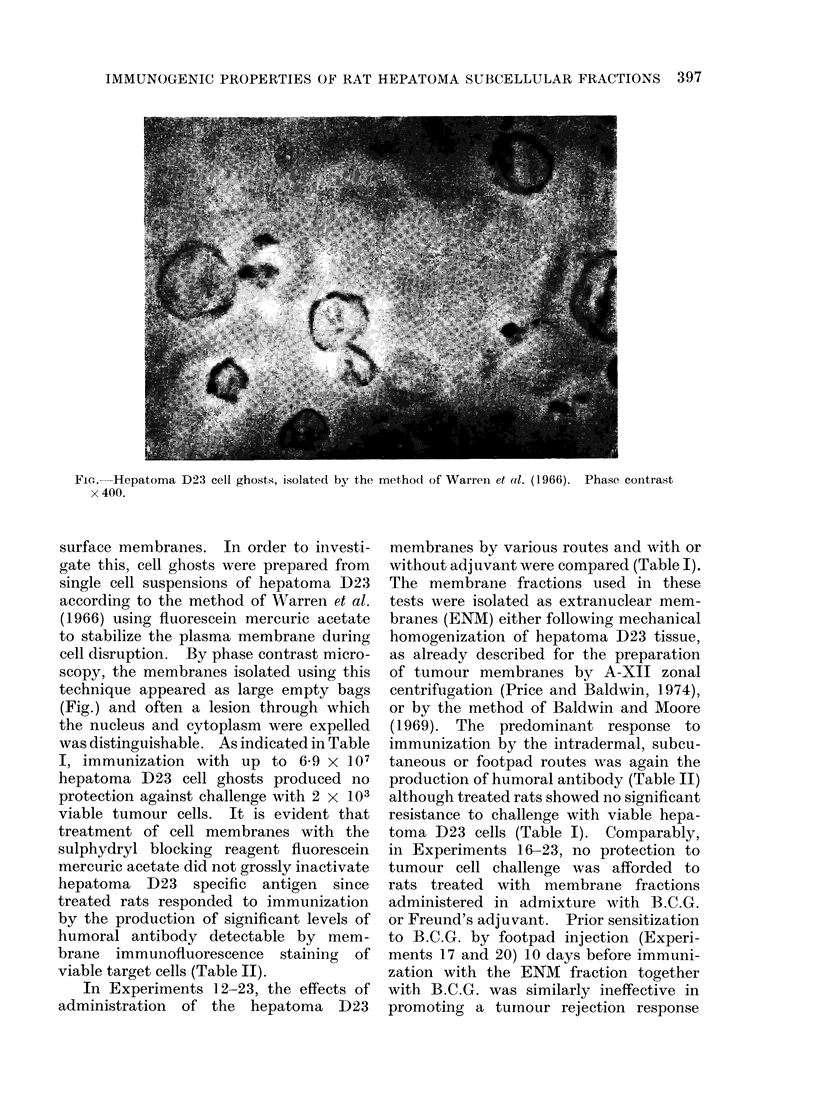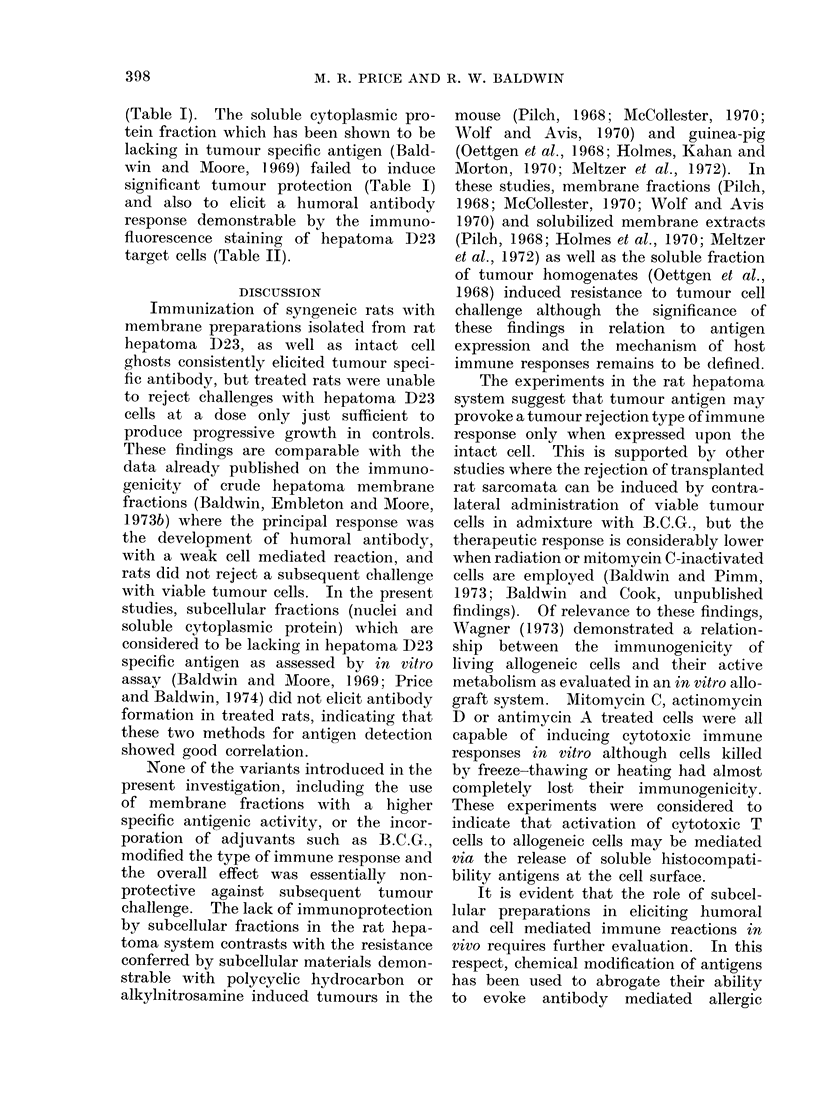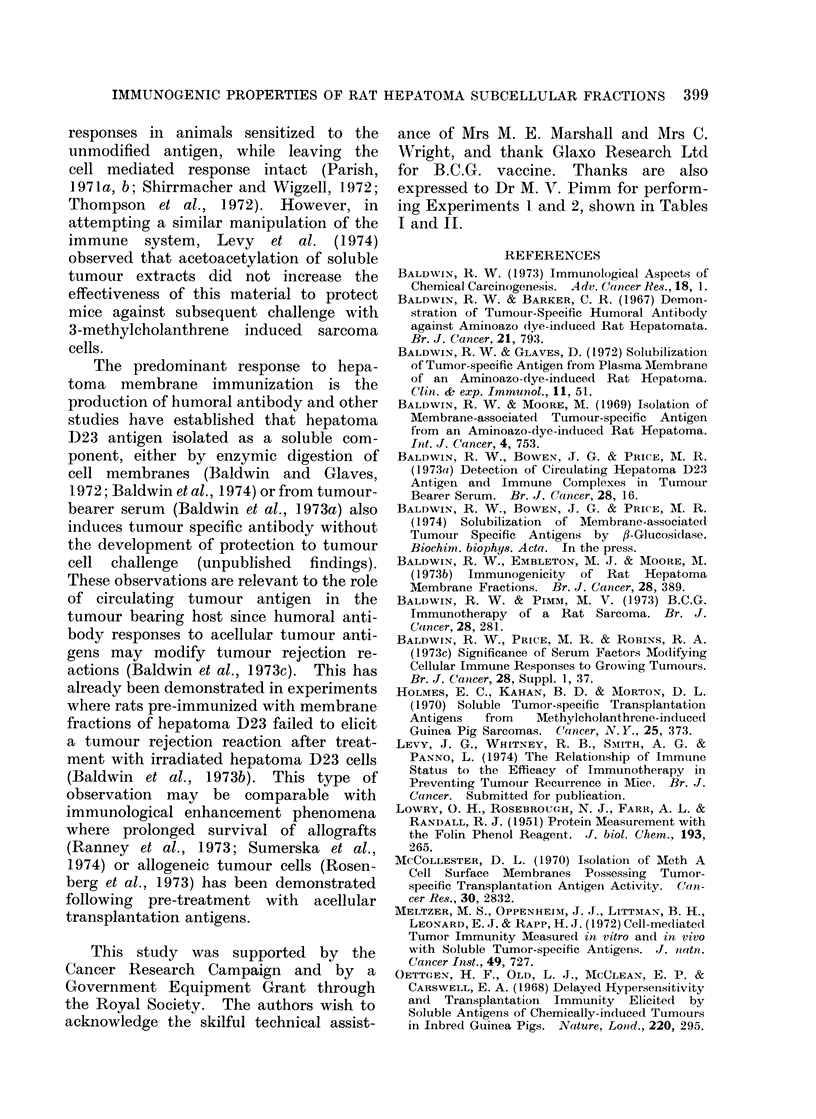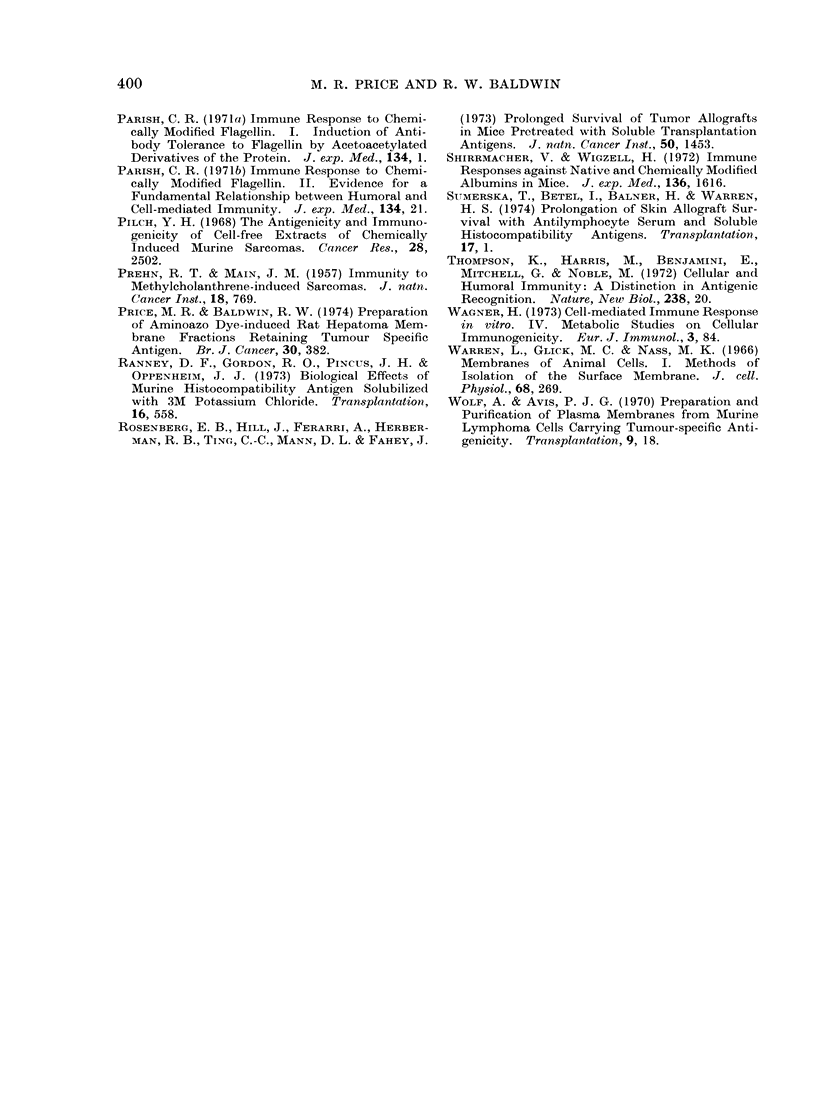# Immunogenic Properties of Rat Hepatoma Subcellular Fractions

**DOI:** 10.1038/bjc.1974.213

**Published:** 1974-11

**Authors:** M. R. Price, R. W. Baldwin

## Abstract

**Images:**


Br. J. Cancer (1974) 30, 394

### IMMUNOGENIC PROPERTIES OF RAT HEPATOMA SUBCELLULAR FRACTIONS

M. R. PRICE AND R. W. BALDWIN

*From the Cancer Research Campaign Laboratories, University of Nottingham, University Park, Nottingham NG7 2RD*

Received 3 July 1974. Accepted 5 July 1974

**Summary.—Subcellular fractions from an aminoazo dye induced rat hepatoma (D23) were examined for their ability to evoke rejection responses in syngeneic hosts to transplanted tumour cells and to induce the production of humoral antibody. Membrane fractions isolated by zonal centrifugation and displaying an increased activity of tumour specific antigen (Price and Baldwin, 1974), as well as crude membrane fractions and purified tumour cell ghosts, all elicited tumour specific antibody demonstrable by membrane immunofluorescence staining of viable hepatoma D23 cells. Tumour cell nuclei or soluble cytoplasmic protein were, however, lacking in this capacity. Resistance to tumour cell challenge was not observed in rats treated with any of the hepatoma D23 subcellular fractions administered by various routes either alone or in admixture with bacterial adjuvants. These findings are relevant to current views that tumour immunity may be more optimally achieved by inoculation of intact (viable or attenuated) tumour cells.**

IMMUNITY to transplanted tumours in syngeneic hosts can be induced by prior treatment of the recipients with tumour cells prevented from progressive growth (reviewed by Baldwin, 1973). This type of immunization has frequently been carried out using tumour cells attenuated by radiation or treatment with cytotoxic drugs, but a more effective response is often achieved following exposure to viable tumour cells. This was originally demonstrated in studies showing the development of tumour immunity following surgical resection of tumour grafts (Prehn and Main, 1957) and is further emphasized by the expression of concomitant immunity in the tumour bearing host (Baldwin, 1973). Also, it has been shown that the tumour immune response elicited with viable 3-methylcholanthrene induced rat sarcoma cells in admixture with B.C.G. is much greater than that produced when radiation attenuated cells are used (Baldwin and Pimm, 1973). These approaches, however, impose serious limitations in designing suitable protocols for immunotherapy since administration of viable tumour cells, even in admixture with bacterial adjuvants, may not be acceptable and there are logistic problems in the use of attenuated tumour cells. From these considerations, it is clearly desirable to employ cell-free tumour antigen preparations and the studies reported here were designed to examine the immunogenicities of isolated aminoazo dye induced rat hepatoma membrane fractions, isolated as described in the previous paper (Price and Baldwin, 1974). These fractions retain tumour specific antigen, as defined by their capacity to interact with tumour specific antibody and, in this report, have been further examined for their capacity both to induce tumour rejection immunity as well as specific humoral antibody responses.

### MATERIALS AND METHODS

*Tumour and tumour cell fractionation.*—Details of the preparation of hepatoma D23 membrane fractions and nuclei are described in the previous paper (Price and Baldwin, 1974).

*Tumour cell ghost preparation.*—Single cell suspensions were prepared from minced hepatoma D23 tissue by repeated treatment with 0·25% trypsin. Tumour cell ghosts were isolated from cell suspensions after stabilization of the cell surface with fluorescein mercuric acetate (Sigma Chemical Co., Kingston-upon-Thames, Surrey) according to the method of Warren, Glick and Nass (1966).

*Preparation of soluble cytoplasmic protein (cell sap).*—Soluble fractions, remaining after centrifugation of 1000 *g* supernatants at 78,000 *g* for 30 min, were dialysed for 4 h against phosphate buffered saline, pH 7·3 (PBS), concentrated against Aquacide II (Calbiochem) and redialysed against PBS for a further 16 h. After centrifugation at 165,000 *g* for 30 min, the supernatant was taken as the soluble cytoplasmic protein (cell sap) fraction, and this was stored at −20°C.

*Immunogenicity tests.*—Syngeneic male rats were immunized against various subcellular fractions by 3 injections at weekly intervals. Adjuvants were mixed at a ratio of 1 : 1 (v/v) with membrane suspensions and 0·2 ml aliquots injected. Freund's complete adjuvant (Difco, Detroit, Michigan) was used and freeze dried B.C.G. (percutaneous) vaccine was supplied by Glaxo Laboratories Ltd (Greenford, Middlesex). Rats were bled from the tail vein 6–10 days after the final injection and serum samples collected from individual rats were stored at −20°C. Subcutaneous challenges with viable hepatoma D23 cells suspended in medium 199 were administered 7–14 days following the final injection of subcellular material. The growth of tumours in immunized rats was compared with that in untreated controls receiving the same tumour cell inoculum.

*Membrane immunofluorescence tests.*—The membrane immunofluorescence test was performed with viable hepatoma D23 cells in suspension as previously described (Baldwin and Barker, 1967).

*Protein determination.*—Protein concentration was determined by the method of Lowry *et al.* (1951).

### RESULTS

### *Immunogenicity of subcellular fractions of hepatoma D23*

Representative tests performed to evaluate the immunogenicity of isolated membrane fractions of hepatoma D23 are summarized in Tables I and II. In these tests, the effectiveness of subcellular fractions in conferring transplantation resistance to tumour cell challenge may be compared with the protective effect elicited by immunization with $\gamma$ irradiated hepatoma D23 cells (Table I). Also, in order to detect any low levels of resistance, challenge doses of between 1 and $5 \times 10^3$ hepatoma cells were chosen since these represent the minimum doses necessary to produce consistent tumour growth in untreated control animals.

Immunization with the antigenic membranes isolated by A-XII or B-XIV zonal centrifugation of hepatoma D23 homogenates induced marked humoral antibody responses essentially comparable with that obtained following immunization with radiation attenuated tumour cells (Table II). No protection, however, was afforded to treated rats following challenge with low doses of hepatoma D23 cells ($1–3 \times 10^3$ cells) and the outgrowth of tumours in immunized and untreated rats was essentially equivalent (Table I). Although the membranes in these tests were isolated from the nuclear sediment (1000 *g* pellets) of hepatoma homogenates, purified nuclei lacked the capacity to induce humoral antibody formation and no significant protection to tumour cell challenge was observed in treated rats (Tables I and II).

Wolf and Avis (1970) have indicated that the size of plasma membrane fragments is critical in the induction of immunity to a carcinogen induced murine lymphoma and greatest protection against viable tumour cell challenge was produced by immunization with almost intact cell ghost preparations rather than fragmented

TABLE I.—*Induction of Immunity to Transplanted Hepatoma D23 by Hepatoma D23 Cells and Subcellular Fractions*

| Expt No. | Immunizing fraction | Total dose (mg protein) | Route of immunization | Tumour rejection tests: Tumour cell challenge ($\times 10^{-3}$) | Tumour takes in: Treated | Tumour takes in: Untreated |
|---|---|---|---|---|---|---|
| 1 | IR cells* | $2\times10^7$ cells | i.p. | 50 | 0/4 | 4/4 |
| 2 | IR cells | $1\times10^7$ cells | i.p. | 500 | 1/4 | 4/4 |
| 3 | A-zonal membrane | 3·9 mg | s.c. | 2 | 5/5 | 5/5 |
| 4 | A-zonal membrane | 27·0 mg | s.c. | 3 | 5/5 | 4/4 |
| 5 | B-zonal membrane | 1·2 mg | s.c. | 1 | 5/5 | 5/5 |
| 6 | B-zonal membrane | 6·3 mg | i.d. | 1 | 5/5 | 5/5 |
| 7 | Nuclei | $6\times10^7$ nuclei | s.c. | 1 | 4/5 | 5/5 |
| 8 | Cell ghosts | $3{\cdot}0\times10^6$ ghosts | i.p | 5 | 5/5 | 6/6 |
| 9 | Cell ghosts | $1{\cdot}2\times10^7$ ghosts | s.c. | 2 | 5/5 | 5/5 |
| 10 | Cell ghosts | $3{\cdot}6\times10^7$ ghosts | s.c. | 2 | 4/5 | 5/5 |
| 11 | Cell ghosts | $6{\cdot}9\times10^7$ ghosts | s.c. | 2 | 5/5 | 5/5 |
| 12 | ENM† | 22·2 mg | i.d. | 1 | 5/5 | 5/5 |
| 13 | ENM+FCA‡ | 22·2 mg | s.c. | 1 | 5/5 | 5/5 |
| 14 | ENM | 22·2 mg | footpad | 1 | 5/5 | 5/5 |
| 15 | ENM | 22·2 mg | s.c. | 1 | 5/5 | 5/5 |
| 16 | ENM+FCA§ | 11·1 mg | s.c. | 1 | 5/5 | 5/5 |
| 17 | ENM+BCG§ | 11·1 mg | s.c. | 1 | 4/5 | 5/5 |
| 18 | ENM+BCG¶ | 11·1 mg | s.c. | 1 | 4/5 | 5/5 |
| 19 | ENM+FCA | 11·1 mg | s.c. | 1 | 5/5 | 5/5 |
| 20 | ENM+BCG§ | 21·0 mg | s.c. | 1 | 5/5 | 5/5 |
| 21 | BCG§ | — | s.c. | 1 | 5/5 | 5/5 |
| 22 | ENM+BCG¶ | 21·0 mg | s.c. | 1 | 5/5 | 5/5 |
| 23 | ENM+FCA | 21·0 mg | s.c. | 1 | 5/5 | 5/5 |
| 24 | Soluble cell sap | 42·0 mg | i.p. | 1 | 4/5 | 5/5 |
| 25 | Soluble cell sap | 98·0 mg | i.p. | 1 | 4/5 | 5/5 |

* IR cells: Rats were immunized by 2 injections of $\gamma$ irradiated (15,000 rad) hepatoma D23 cells.

† ENM: Extranuclear membranes; in Experiments 12–19 ENM fractions were prepared by the method of Baldwin and Moore (1969) and in Experiments 20–23 ENM fractions were isolated as described by Price and Baldwin (1974) using procedures developed for A-XII zonal centrifugation.

‡ FCA: Freund's complete adjuvant.

§ Rats were pre-sensitized to the adjuvant by footpad injection 0·1 ml FCA or 0·1 ml FCA containing 0·5 mg moist weight of B.C.G. 10 days before the first injection. For immunization, membrane suspensions were mixed at a ratio of 1 : 1 with the sensitizing adjuvant.

¶ 0·5 mg moist weight of B.C.G. in 0·1 ml FCA were injected in admixture with ENM suspensions.

TABLE II.—*Humoral Antibody Response Evoked by Cells and Subcellular Fractions of Hepatoma D23*

| Expt No. | Immunizing fraction | Total dose (mg protein) | Route of immunization | Serum antibody*: No. positive / No. tested | Serum antibody*: Fluorescence index |
|---|---|---|---|---|---|
| 1 | IR cells† | $2\times10^7$ cells | i.p. | 4/4 | 0·34–0·55 |
| 2 | IR cells | $1\times10^7$ cells | i.p. | 2/4 | 0·25–0·38 |
| 3 | A-zonal membrane | 3·9 mg | s.c. | 5/5 | 0·45–0·55 |
| 4 | A-zonal membrane | 27·0 mg | s.c. | 5/5 | 0·51–0·63 |
| 5 | B-zonal membrane | 1·2 mg | s.c. | 5/5 | 0·47–0·58 |
| 6 | B-zonal membrane | 6·3 mg | i.d. | 5/5 | 0·62–0·65 |
| 7 | Nuclei | $6\times10^7$ nuclei | s.c. | 0/5 | 0·00–0·07 |
| 8 | Cell ghosts | $3{\cdot}0\times10^6$ ghosts | i.p. | 4/5 | 0·24–0·55 |
| 12 | ENM‡ | 22·2 mg | i.d. | 4/5 | 0·29–0·48 |
| 13 | ENM+FCA | 22·2 mg | s.c. | 5/5 | 0·32–0·46 |
| 14 | ENM | 22·2 mg | footpad | 4/5 | 0·29–0·51 |
| 15 | ENM | 22·2 mg | s.c. | 5/5 | 0·33–0·66 |
| 24 | Soluble cell sap | 42·0 mg | i.p. | 0/5 | 0·00–0·08 |

* Serum antibody was detected using the indirect membrane immunofluorescence test as described by Baldwin and Barker (1967). Serum fluorescence indices of 0·3 or greater represents a positive reaction.

† IR cells: Rats were immunized by 2 injections of $\gamma$ irradiated (15,000 rad) hepatoma D23 cells.

‡ ENM: Extranuclear membranes were prepared by the method of Baldwin and Moore (1969).

FIG.—Hepatoma D23 cell ghosts, isolated by the method of Warren *et al.* (1966). Phase contrast ×400.

surface membranes. In order to investigate this, cell ghosts were prepared from single cell suspensions of hepatoma D23 according to the method of Warren *et al.* (1966) using fluorescein mercuric acetate to stabilize the plasma membrane during cell disruption. By phase contrast microscopy, the membranes isolated using this technique appeared as large empty bags (Fig.) and often a lesion through which the nucleus and cytoplasm were expelled was distinguishable. As indicated in Table I, immunization with up to $6 \cdot 9 \times 10^7$ hepatoma D23 cell ghosts produced no protection against challenge with $2 \times 10^3$ viable tumour cells. It is evident that treatment of cell membranes with the sulphydryl blocking reagent fluorescein mercuric acetate did not grossly inactivate hepatoma D23 specific antigen since treated rats responded to immunization by the production of significant levels of humoral antibody detectable by membrane immunofluorescence staining of viable target cells (Table II).

In Experiments 12–23, the effects of administration of the hepatoma D23 membranes by various routes and with or without adjuvant were compared (Table I). The membrane fractions used in these tests were isolated as extranuclear membranes (ENM) either following mechanical homogenization of hepatoma D23 tissue, as already described for the preparation of tumour membranes by A-XII zonal centrifugation (Price and Baldwin, 1974), or by the method of Baldwin and Moore (1969). The predominant response to immunization by the intradermal, subcutaneous or footpad routes was again the production of humoral antibody (Table II) although treated rats showed no significant resistance to challenge with viable hepatoma D23 cells (Table I). Comparably, in Experiments 16–23, no protection to tumour cell challenge was afforded to rats treated with membrane fractions administered in admixture with B.C.G. or Freund's adjuvant. Prior sensitization to B.C.G. by footpad injection (Experiments 17 and 20) 10 days before immunization with the ENM fraction together with B.C.G. was similarly ineffective in promoting a tumour rejection response

(Table I). The soluble cytoplasmic protein fraction which has been shown to be lacking in tumour specific antigen (Baldwin and Moore, 1969) failed to induce significant tumour protection (Table I) and also to elicit a humoral antibody response demonstrable by the immunofluorescence staining of hepatoma D23 target cells (Table II).

### DISCUSSION

Immunization of syngeneic rats with membrane preparations isolated from rat hepatoma D23, as well as intact cell ghosts consistently elicited tumour specific antibody, but treated rats were unable to reject challenges with hepatoma D23 cells at a dose only just sufficient to produce progressive growth in controls. These findings are comparable with the data already published on the immunogenicity of crude hepatoma membrane fractions (Baldwin, Embleton and Moore, 1973*b*) where the principal response was the development of humoral antibody, with a weak cell mediated reaction, and rats did not reject a subsequent challenge with viable tumour cells. In the present studies, subcellular fractions (nuclei and soluble cytoplasmic protein) which are considered to be lacking in hepatoma D23 specific antigen as assessed by *in vitro* assay (Baldwin and Moore, 1969; Price and Baldwin, 1974) did not elicit antibody formation in treated rats, indicating that these two methods for antigen detection showed good correlation.

None of the variants introduced in the present investigation, including the use of membrane fractions with a higher specific antigenic activity, or the incorporation of adjuvants such as B.C.G., modified the type of immune response and the overall effect was essentially nonprotective against subsequent tumour challenge. The lack of immunoprotection by subcellular fractions in the rat hepatoma system contrasts with the resistance conferred by subcellular materials demonstrable with polycyclic hydrocarbon or alkylnitrosamine induced tumours in the mouse (Pilch, 1968; McCollester, 1970; Wolf and Avis, 1970) and guinea-pig (Oettgen *et al.*, 1968; Holmes, Kahan and Morton, 1970; Meltzer *et al.*, 1972). In these studies, membrane fractions (Pilch, 1968; McCollester, 1970; Wolf and Avis 1970) and solubilized membrane extracts (Pilch, 1968; Holmes *et al.*, 1970; Meltzer *et al.*, 1972) as well as the soluble fraction of tumour homogenates (Oettgen *et al.*, 1968) induced resistance to tumour cell challenge although the significance of these findings in relation to antigen expression and the mechanism of host immune responses remains to be defined.

The experiments in the rat hepatoma system suggest that tumour antigen may provoke a tumour rejection type of immune response only when expressed upon the intact cell. This is supported by other studies where the rejection of transplanted rat sarcomata can be induced by contralateral administration of viable tumour cells in admixture with B.C.G., but the therapeutic response is considerably lower when radiation or mitomycin C-inactivated cells are employed (Baldwin and Pimm, 1973; Baldwin and Cook, unpublished findings). Of relevance to these findings, Wagner (1973) demonstrated a relationship between the immunogenicity of living allogeneic cells and their active metabolism as evaluated in an *in vitro* allograft system. Mitomycin C, actinomycin D or antimycin A treated cells were all capable of inducing cytotoxic immune responses *in vitro* although cells killed by freeze–thawing or heating had almost completely lost their immunogenicity. These experiments were considered to indicate that activation of cytotoxic T cells to allogeneic cells may be mediated *via* the release of soluble histocompatibility antigens at the cell surface.

It is evident that the role of subcellular preparations in eliciting humoral and cell mediated immune reactions *in vivo* requires further evaluation. In this respect, chemical modification of antigens has been used to abrogate their ability to evoke antibody mediated allergic responses in animals sensitized to the unmodified antigen, while leaving the cell mediated response intact (Parish, 1971*a*, *b*; Shirrmacher and Wigzell, 1972; Thompson *et al.*, 1972). However, in attempting a similar manipulation of the immune system, Levy *et al.* (1974) observed that acetoacetylation of soluble tumour extracts did not increase the effectiveness of this material to protect mice against subsequent challenge with 3-methylcholanthrene induced sarcoma cells.

The predominant response to hepatoma membrane immunization is the production of humoral antibody and other studies have established that hepatoma D23 antigen isolated as a soluble component, either by enzymic digestion of cell membranes (Baldwin and Glaves, 1972; Baldwin *et al.*, 1974) or from tumour-bearer serum (Baldwin *et al.*, 1973*a*) also induces tumour specific antibody without the development of protection to tumour cell challenge (unpublished findings). These observations are relevant to the role of circulating tumour antigen in the tumour bearing host since humoral antibody responses to acellular tumour antigens may modify tumour rejection reactions (Baldwin *et al.*, 1973*c*). This has already been demonstrated in experiments where rats pre-immunized with membrane fractions of hepatoma D23 failed to elicit a tumour rejection reaction after treatment with irradiated hepatoma D23 cells (Baldwin *et al.*, 1973*b*). This type of observation may be comparable with immunological enhancement phenomena where prolonged survival of allografts (Ranney *et al.*, 1973; Sumerska *et al.*, 1974) or allogeneic tumour cells (Rosenberg *et al.*, 1973) has been demonstrated following pre-treatment with acellular transplantation antigens.

This study was supported by the Cancer Research Campaign and by a Government Equipment Grant through the Royal Society. The authors wish to acknowledge the skilful technical assistance of Mrs M. E. Marshall and Mrs C. Wright, and thank Glaxo Research Ltd for B.C.G. vaccine. Thanks are also expressed to Dr M. V. Pimm for performing Experiments 1 and 2, shown in Tables I and II.

### REFERENCES

BALDWIN, R. W. (1973) Immunological Aspects of Chemical Carcinogenesis. *Adv. Cancer Res.*, **18**, 1.

BALDWIN, R. W. & BARKER, C. R. (1967) Demonstration of Tumour-Specific Humoral Antibody against Aminoazo dye-induced Rat Hepatomata. *Br. J. Cancer*, **21**, 793.

BALDWIN, R. W. & GLAVES, D. (1972) Solubilization of Tumor-specific Antigen from Plasma Membrane of an Aminoazo-dye-induced Rat Hepatoma. *Clin. & exp. Immunol.*, **11**, 51.

BALDWIN, R. W. & MOORE, M. (1969) Isolation of Membrane-associated Tumour-specific Antigen from an Aminoazo-dye-induced Rat Hepatoma. *Int. J. Cancer*, **4**, 753.

BALDWIN, R. W., BOWEN, J. G. & PRICE, M. R. (1973*a*) Detection of Circulating Hepatoma D23 Antigen and Immune Complexes in Tumour Bearer Serum. *Br. J. Cancer*, **28**, 16.

BALDWIN, R. W., BOWEN, J. G. & PRICE, M. R. (1974) Solubilization of Membrane-associated Tumour Specific Antigens by $\beta$-Glucosidase. *Biochim. biophys. Acta.* In the press.

BALDWIN, R. W., EMBLETON, M. J. & MOORE, M. (1973*b*) Immunogenicity of Rat Hepatoma Membrane Fractions. *Br. J. Cancer*, **28**, 389.

BALDWIN, R. W. & PIMM, M. V. (1973) B.C.G. Immunotherapy of a Rat Sarcoma. *Br. J. Cancer*, **28**, 281.

BALDWIN, R. W., PRICE, M. R. & ROBINS, R. A. (1973*c*) Significance of Serum Factors Modifying Cellular Immune Responses to Growing Tumours. *Br. J. Cancer*, **28**, Suppl. 1, 37.

HOLMES, E. C., KAHAN, B. D. & MORTON, D. L. (1970) Soluble Tumor-specific Transplantation Antigens from Methylcholanthrene-induced Guinea Pig Sarcomas. *Cancer, N.Y.*, **25**, 373.

LEVY, J. G., WHITNEY, R. B., SMITH, A. G. & PANNO, L. (1974) The Relationship of Immune Status to the Efficacy of Immunotherapy in Preventing Tumour Recurrence in Mice. *Br. J. Cancer.* Submitted for publication.

LOWRY, O. H., ROSEBROUGH, N. J., FARR, A. L. & RANDALL, R. J. (1951) Protein Measurement with the Folin Phenol Reagent. *J. biol. Chem.*, **193**, 265.

MCCOLLESTER, D. L. (1970) Isolation of Meth A Cell Surface Membranes Possessing Tumor-specific Transplantation Antigen Activity. *Cancer Res.*, **30**, 2832.

MELTZER, M. S., OPPENHEIM, J. J., LITTMAN, B. H., LEONARD, E. J. & RAPP, H. J. (1972) Cell-mediated Tumor Immunity Measured *in vitro* and *in vivo* with Soluble Tumor-specific Antigens. *J. natn. Cancer Inst.*, **49**, 727.

OETTGEN, H. F., OLD, L. J., MCCLEAN, E. P. & CARSWELL, E. A. (1968) Delayed Hypersensitivity and Transplantation Immunity Elicited by Soluble Antigens of Chemically-induced Tumours in Inbred Guinea Pigs. *Nature, Lond.*, **220**, 295.

PARISH, C. R. (1971*a*) Immune Response to Chemically Modified Flagellin. I. Induction of Antibody Tolerance to Flagellin by Acetoacetylated Derivatives of the Protein. *J. exp. Med.*, **134**, 1.

PARISH, C. R. (1971*b*) Immune Response to Chemically Modified Flagellin. II. Evidence for a Fundamental Relationship between Humoral and Cell-mediated Immunity. *J. exp. Med.*, **134**, 21.

PILCH, Y. H. (1968) The Antigenicity and Immunogenicity of Cell-free Extracts of Chemically Induced Murine Sarcomas. *Cancer Res.*, **28**, 2502.

PREHN, R. T. & MAIN, J. M. (1957) Immunity to Methylcholanthrene-induced Sarcomas. *J. natn. Cancer Inst.*, **18**, 769.

PRICE, M. R. & BALDWIN, R. W. (1974) Preparation of Aminoazo Dye-induced Rat Hepatoma Membrane Fractions Retaining Tumour Specific Antigen. *Br. J. Cancer*, **30**, 382.

RANNEY, D. F., GORDON, R. O., PINCUS, J. H. & OPPENHEIM, J. J. (1973) Biological Effects of Murine Histocompatibility Antigen Solubilized with 3M Potassium Chloride. *Transplantation*, **16**, 558.

ROSENBERG, E. B., HILL, J., FERARRI, A., HERBERMAN, R. B., TING, C.-C., MANN, D. L. & FAHEY, J. (1973) Prolonged Survival of Tumor Allografts in Mice Pretreated with Soluble Transplantation Antigens. *J. natn. Cancer Inst.*, **50**, 1453.

SHIRRMACHER, V. & WIGZELL, H. (1972) Immune Responses against Native and Chemically Modified Albumins in Mice. *J. exp. Med.*, **136**, 1616.

SUMERSKA, T., BETEL, I., BALNER, H. & WARREN, H. S. (1974) Prolongation of Skin Allograft Survival with Antilymphocyte Serum and Soluble Histocompatibility Antigens. *Transplantation*, **17**, 1.

THOMPSON, K., HARRIS, M., BENJAMINI, E., MITCHELL, G. & NOBLE, M. (1972) Cellular and Humoral Immunity: A Distinction in Antigenic Recognition. *Nature, New Biol.*, **238**, 20.

WAGNER, H. (1973) Cell-mediated Immune Response *in vitro*. IV. Metabolic Studies on Cellular Immunogenicity. *Eur. J. Immunol.*, **3**, 84.

WARREN, L., GLICK, M. C. & NASS, M. K. (1966) Membranes of Animal Cells. I. Methods of Isolation of the Surface Membrane. *J. cell. Physiol.*, **68**, 269.

WOLF, A. & AVIS, P. J. G. (1970) Preparation and Purification of Plasma Membranes from Murine Lymphoma Cells Carrying Tumour-specific Antigenicity. *Transplantation*, **9**, 18.